# Health-Related Quality of Life in Long-Term Survivors of Relapsed Childhood Acute Lymphoblastic Leukemia

**DOI:** 10.1371/journal.pone.0038015

**Published:** 2012-05-25

**Authors:** Stefan Essig, Nicolas X. von der Weid, Marie-Pierre F. Strippoli, Cornelia E. Rebholz, Gisela Michel, Corina S. Rueegg, Felix K. Niggli, Claudia E. Kuehni, for the Swiss Pediatric Oncology Group (SPOG)

**Affiliations:** 1 Swiss Childhood Cancer Registry, Institute of Social and Preventive Medicine, University of Bern, Bern, Switzerland; 2 Paediatric Hematology-Oncology Unit, Centre Hospitalier Universitaire Vaudois, Lausanne, Switzerland; 3 Department of Oncology, University Children’s Hospital Zurich, Zurich, Switzerland; University of Massachusetts Medical School, United States of America

## Abstract

**Background:**

Relapses occur in about 20% of children with acute lymphoblastic leukemia (ALL). Approximately one-third of these children can be cured. Their risk for late effects is high because of intensified treatment, but their health-related quality of life (HRQOL) was largely unmeasured. Our aim was to compare HRQOL of ALL survivors with the general population, and of relapsed with non-relapsed ALL survivors.

**Methodology/Principal Findings:**

As part of the Swiss Childhood Cancer Survivor Study (SCCSS) we sent a questionnaire to all ALL survivors in Switzerland who had been diagnosed between 1976–2003 at age <16 years, survived ≥5 years, and were currently aged ≥16 years. HRQOL was assessed with the Short Form-36 (SF-36), which measures four aspects of physical health and four aspects of mental health. A score of 50 corresponded to the mean of a healthy reference population. We analyzed data from 457 ALL survivors (response: 79%). Sixty-one survivors had suffered a relapse. Compared to the general population, ALL survivors reported similar or higher HRQOL scores on all scales. Survivors with a relapse scored lower in general health perceptions (51.6) compared to those without (55.8;p=0.005), but after adjusting for self-reported late effects, this difference disappeared.

**Conclusion/Significance:**

Compared to population norms, ALL survivors reported good HRQOL, even after a relapse. However, relapsed ALL survivors reported poorer general health than non-relapsed. Therefore, we encourage specialists to screen for poor general health in survivors after a relapse and, when appropriate, specifically seek and treat underlying late effects. This will help to improve patients’ HRQOL.

## Introduction

Acute lymphoblastic leukemia (ALL) is the most common cancer in children younger than 15 years of age, accounting for about 28% of malignancies in the pediatric population [1], [2]. Therapy has dramatically improved over the last decades and overall survival for children with ALL is now 85% [3], [4]. Nevertheless, about 15–20% of children with ALL suffer from relapse [5]. Current salvage protocols result in cures for 37% of these patients, who undergo intensified chemotherapy, often including central nervous system irradiation and/or stem cell transplantation [6]. The disease and its treatment put ALL survivors are at risk for somatic and neuropsychological late effects and second malignancies [7]–[11]. This is particularly true of survivors of relapsed ALL, who more frequently develop more severe chronic medical conditions, affecting more organ systems, than non-relapsed ALL survivors. Late effects are observed in cardiac, endocrine, neurologic, renal, and visual systems [12], [13].

We know a good deal about late effects, but less is known about health-related quality of life (HRQOL) of relapsed and non-relapsed ALL survivors [14]–[17]. HRQOL assesses subjectively perceived functioning [18], [19] and is a multidimensional construct of physical, psychological and social well being and the capacity to perform the activities of daily life. Three previous studies have compared HRQOL of ALL survivors with population norms and reported similar or slightly lower HRQOL in survivors [20]–[22], but they did not distinguish between patients who had relapsed and those who had not. Studies of a cohort of childhood cancer survivors with different diagnoses reported that relapse had no effect on HRQOL [14], [15], but others studies found more than one treatment series (as a proxy for relapse) to be independently associated with poorer HRQOL [16]. A small hospital-based study in Finland found better HRQOL in relapsed ALL survivors compared to ALL survivors without relapse and to healthy controls [17].

Our goal was to better understand the role that relapse plays in the HRQOL of patients who have survived the complex course of ALL and its treatment. Therefore, we compared HRQOL of ALL survivors with the general population (1) and of relapsed and non-relapsed ALL survivors, accounting for late effects (2).

## Methods

### Ethics Statement

Since 2004, all patients and their families give informed consent at the time of cancer diagnosis for their data to be included in the Swiss Childhood Cancer Registry (SCCR) and used for research. Patients who had been diagnosed in the early years of the registry received the information retrospectively and could object to their inclusion in the registry (right of veto). This procedure was decided by the Swiss Federal Commission of Experts for Professional Secrecy in Medical Research when granting the general cancer registry permission to the SCCR, and was endorsed by the ethics committee of the canton of Bern. Similarly, the questionnaire survey of the Swiss Childhood Cancer Survivor Study was approved by the ethics committee of the canton of Bern. When returning the questionnaire, cancer survivors consented that their data are used for research. All information regarding individuals was made anonymous to investigators prior to analysis.

### Sample and Procedure

The Swiss Childhood Cancer Survivor Study (SCCSS) is a population-based long-term follow-up study of all childhood cancer patients registered in the Swiss Childhood Cancer Registry (SCCR), diagnosed 1976–2003, who survived for at least 5 years [7], [23]. The SCCR includes all children and adolescents in Switzerland diagnosed with leukemia, lymphoma, central nervous system tumors, malignant solid tumors or Langerhans cell histiocytosis before age 16 years [2], [24].

Eligible participants were traced with an extensive address search procedure. Between 2007 and 2010, survivors with identified addresses received an information letter from their former pediatric oncology clinic, followed in two weeks by a questionnaire that included a pre-paid return envelope. Four weeks later, non-responders received a reminder letter, and a phone call six weeks later. Letters and questionnaires were written in the three national languages: German, French and Italian. For the current analyses we included only ALL survivors at least 16 years old at the time of the study, who had had no relapse and no second malignancies in the five years before survey. For the current analyses we included only ALL survivors at least 16 years old at the time of the study, who had had no relapse and no second malignancies in the five years before survey. This was done because we wanted to assess quality of life in state of relative health not during treatment of a relapse or second tumor.

### Measurements

#### a) Health-Related Quality of Life

The SCCSS used an extensive questionnaire similar to that used in US and British childhood cancer survivors studies [25], [26]. It included the Short Form-36 (SF-36) to measure HRQOL [27]. This instrument SF-36 is psychometrically tested and available in several languages [19], [27], [28]. The SF-36 has been successfully used in samples of long-term childhood cancer survivors [20], [22], and is valid and reliable [29]. It consists of 36 questions that can be aggregated into eight scales: physical functioning, role limitations due to physical health, bodily pain, general health perceptions, vitality, social functioning, role limitations due to emotional problems, and mental health (**Table S1**). We standardized the survivors’ scores as T-scores according to separate German, French and Italian population norms [30]–[32], using a general population mean of 50 and standard deviation of 10. A public-use file from the German Federal Health Survey (N=6964) allowed us to select a subgroup similar in age and gender distribution to the survivors [33]. Higher scores indicate better HRQOL.

#### b) Clinical and Socio-Demographic Information

Information on baseline demographics and prospectively collected medical information on survivor diagnosis and treatment was extracted from the Swiss Childhood Cancer Registry: current age; gender; age at diagnosis; time since diagnosis; chemo- and radiotherapy (chemotherapy: without radiotherapy, may have surgery/radiotherapy: with or without chemotherapy or surgery); bone marrow transplantation; duration of therapy; and relapse status. Therapy variables included treatment both for initial and relapsed ALL. This information was extracted from the SCCSS questionnaire: having a partner (yes/no); education; and self-reported late effects. Education was divided into four categories according to the Swiss Census: compulsory schooling; vocational training; upper secondary education; and, university education [34]. Survivors were asked whether they experienced any late effects of their cancer or treatment (yes/no) to assess late effects. Late effects were defined as adverse long-term outcomes of cancer or treatment, including somatic and psychological problems, as described by the survivors in open format. Relapse was not considered a late effect.

### Statistical Analyses

#### a) Quality of HRQOL Data

We determined the quality of HRQOL data by calculating missing values per item, item-scale internal consistency, and Cronbach’s alpha.

#### b) Comparison with Norm Population and within Survivor Population

We calculated the ALL survivors’ (all, relapsed only, non-relapsed only) mean t-scores for each scale to find out if they were within one standard deviation of the population norms (Aim 1). HRQOL within one standard deviation of the general population was considered “normal” [22].

We also compared HRQOL of relapsed with non-relapsed ALL survivors (Aim 2), performing univariable and multivariable linear regressions for each SF-36 scale (dependent variables). Relapse status was the independent variable in univariable analysis. We added more variables in two steps in multivariable analysis:

- Step 1, baseline model, adjusting for possible confounders (variables: gender, current age, time since diagnosis);
- Step 2, extended models, adjusting for additional variables to investigate associations between relapse and HRQOL:
  - Social model (having a partner, education)
  - Therapy model (chemo−/radiotherapy, bone marrow transplantation, duration of therapy)
  - Late effects model (self-reported late effects)
  - Full model (adjusting for all variables)

We performed in-depth analysis of SF-36 scales associated with relapse status checking the answers that aggregated into the respective scales.

#### c) Sensitivity Analyses

In sensitivity analyses, we used the French and Italian population norms instead of the German.

Analyses were carried out using the software package STATA version 12 (Stata Corporation, Austin, TX, USA).

## Results

**Figure 1:**
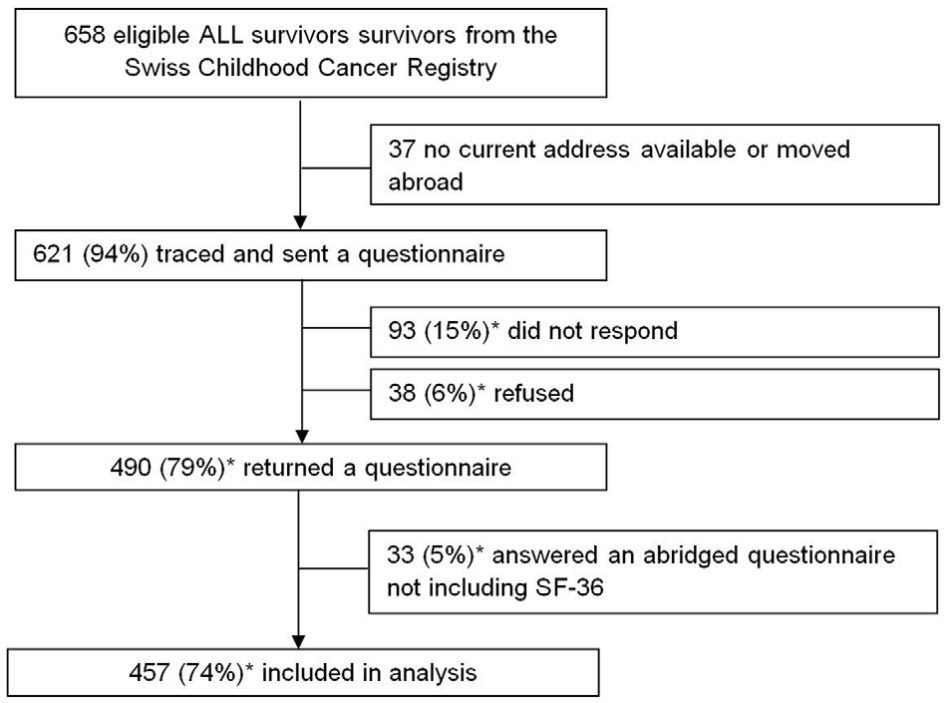
Participant status of acute lymphoblastic leukemia survivors in the Swiss Childhood Cancer Survivor Study. As of May 19, 2011. *of those traced and sent questionnaire. ALL: acute lymphoblastic leukemia; SF-36: Short Form-36.

We successfully traced addresses for 621 of 658 eligible ALL survivors (**Figure 1**). Of those, 490 (79% of contacted) returned the questionnaire. We excluded from the analysis survivors who answered an abridged questionnaire without an SF-36 (n=33), which limited our sample to 457 survivors (74% of contacted).

### Characteristics of the Study Population

Participants (n=457) were more often female (50% vs. 35%, p=0.001), and more often treated with a bone marrow transplantation (11% vs. 4%, p=0.016) than those who did not reply or completed only an abridged questionnaire (n=164) (**Table 1**). There was no difference in current age, age at diagnosis, time since diagnosis, chemo−/radiotherapy, duration of therapy or relapse status.

**Table 1:** Characteristics of non-participants and participants.

		Non-participants		Participants							
		All (n=164)		All (n=457)			Relapse (n=61)		Non-Relapse (n=396)		
		n	%	n	%	p^a^	n	%	n	%	p^b^
Gender	Male	107	65.2	229	50.1		36	59.0	193	48.7	
	Female	57	34.8	228	49.9	0.001	25	41.0	203	51.3	0.135
Current age	16–24.9 y	75	45.7	236	51.6		26	42.6	210	53.0	
	25–29.9 y	31	18.9	110	24.1		12	19.7	98	24.7	
	30–34.9 y	28	17.1	62	13.6		12	19.7	50	12.6	
	≥35 y	25	15.2	49	10.7	0.057	11	18.0	38	9.6	0.015
Having a partner	No		n/a	234	51.2		32	52.5	202	51.0	
	Yes		n/a	209	45.7	n/a	27	44.3	182	46.0	0.815
Education	Compulsory schooling		n/a	114	24.9		13	21.3	101	25.5	
	Vocational training		n/a	179	39.2		29	47.5	150	37.9	
	Upper secondary education^c^		n/a	88	19.3		11	18.0	77	19.4	
	University education		n/a	58	12.7	n/a	7	11.5	51	12.9	0.966
Age at diagnosis	0–4.9 y	72	43.9	227	49.7		29	47.5	198	50.0	
	5–9.9 y	56	34.1	136	29.8		20	32.8	116	29.3	
	≥10 y	36	22.0	94	20.6	0.317	12	19.7	82	20.7	0.895
Time since diagnosis	5–14.9 y	44	26.8	125	27.4		8	13.1	117	29.5	
	15–24.9 y	52	31.7	239	52.3		32	52.5	207	52.3	
	≥25	38	23.2	93	20.4	0.717	21	34.4	72	18.2	0.001
Chemo−/Radiotherapy	Chemotherapy^d^	104	63.4	312	68.3		8	13.1	304	76.8	
	Radiotherapy^e^	60	36.6	145	31.7	0.257	53	86.9	92	23.2	<0.001
	No	157	95.7	409	89.5		41	67.2	368	92.9	
	Yes	7	4.3	48	10.5	0.016	20	32.8	28	7.1	<0.001
Duration of therapy	≤2 y	101	61.6	305	66.7		18	29.5	287	72.5	
	>3 y	42	25.6	119	26.0	0.765	42	68.9	77	19.4	<0.001
Self-reported late effects	No		n/a	334	73.1		26	42.6	308	77.8	
	Yes		n/a	119	26.0	n/a	35	57.4	84	21.2	<0.001
Relapse status	No	133	81.1	396	86.7		0	0	396	100	
	Yes	31	18.9	61	13.3	0.086	61	100	0	0	n/a

Abbreviation: n/a, not applicable.

a all non-participants vs. all participants; p-values calculated from chi-square statistics.

b relapsed vs. non-relapsed participants; p-values calculated from chi-square statistics.

c includes high school, teachers training colleges, technical colleges and upper vocational education.

d without radiotherapy, may have surgery.

e with or without chemotherapy or surgery.

Compared to survivors of non-relapsed ALL (n=396), survivors of relapsed ALL (n=61) were older, diagnosed earlier, more likely to report late effects, and more likely to have received radiotherapy, bone marrow transplant or treatment lasting 3 years or longer. There was no difference in gender, relationship status, education, or age at diagnosis.

### Quality of HRQOL Data

Data on HRQOL was nearly complete, missing values per item ranging from 1–2%. The correlation between each item with its hypothesized scale exceeded the suggested standard of 0.40 for satisfactory item-consistency (0.44–0.82). Cronbach’s alpha was high for all scales (0.78–0.90) suggesting good reliability.

### Comparison with Norm Population

Compared with the German population norm, survivors’ scores on all HRQOL scales were similar or higher (**Table 2**). Mean scores for all SF-36 scales were within one standard deviation of the norm no matter the relapse status: Relapsed ALL survivors scored lowest in the scale “role emotional” (47.4) and highest in the scale “bodily pain” (57.1); non-Relapsed ALL survivors scored lowest score in “role emotional” (49.1) and highest in “vitality” (57.4).

**Table 2:** SF-36 scales, in all survivors and comparing those with and without relapse.

		Unadjusted				Adjusted, full model^a^			
		All(n=457)	Non-relapse (n=396)	Relapse (n=61)	p	All(n=457)	Non-relapse (n=396)	Relapse (n=61)	p^b^
Physical functioning	Mean	52.0	52.1	50.8	0.126	52.0	51.8	53.4	0.219
	95CI	[51.2–52.7]	[51.4–52.9]	[48.8–52.8]		[51.3–52.8]	[51.0–52.6]	[51.1–55.7]	
Role physical	Mean	51.0	51.1	49.8	0.132	51.0	51.0	51.2	0.817
	95CI	[50.3–51.6]	[50.5–51.8]	[47.9–51.7]		[50.4–51.6]	[50.3–51.7]	[49.2–53.3]	
Bodily pain	Mean	57.1	57.1	57.1	0.781	57.1	56.9	58.3	0.245
	95CI	[56.5–57.8]	[56.4–57.9]	[55.2–58.9]		[56.4–57.8]	[56.1–57.7]	[56.1–60.5]	
General health	Mean	55.3	55.8	51.6	0.005	55.4	55.8	53.0	0.135
	95CI	[54.2–56.3]	[54.7–57.0]	[48.6–54.7]		[54.3–56.5]	[54.6–57.0]	[49.6–56.4]	
Vitality	Mean	57.0	57.4	54.3	0.087	57.0	57.1	56.1	0.606
	95CI	[55.8–58.1]	[56.1–58.6]	[50.7–57.8]		[55.8–58.1]	[55.8–58.4]	[52.3–59.8]	
Social functioning	Mean	50.9	51.1	49.3	0.113	50.9	51.0	50.6	0.799
	95CI	[50.0–51.8]	[50.2–52.1]	[46.2–52.4]		[50.0–51.9]	[50.0–52.0]	[47.6–53.5]	
Role emotional	Mean	48.8	49.1	47.4	0.230	48.8	48.8	48.7	0.931
	95CI	[48.0–49.6]	[48.2–49.9]	[44.7–50.1]		[47.9–49.6]	[47.9–49.7]	[46.0–51.3]	
Mental health	Mean	54.0	54.2	52.8	0.488	54.0	54.0	53.9	0.989
	95CI	[53.0–55.0]	[53.1–55.2]	[49.9–55.7]		[52.9–55.0]	[52.8–55.1]	[50.7–57.2]	

Abbreviation: 95CI, 95% confidence interval.

a adjusted for gender, current age, time since diagnosis, having a partner, education, chemo−/radiotherapy, bone marrow transplantation, duration of therapy, and self-reported late effects.

b p-values calculated from likelihood-ratio tests.

### Comparison within Survivor Population

In a comparison of relapsed and non-relapsed ALL survivor HRQOL, those who relapsed tended to be low in some SF-36 scales, but on only one scale was relapse significantly associated in univariable analysis. Survivors of relapsed ALL had a lower “general health” score than survivors of non-relapsed ALL (51.6 vs. 55.8, p=0.005).

**Figure 2:**
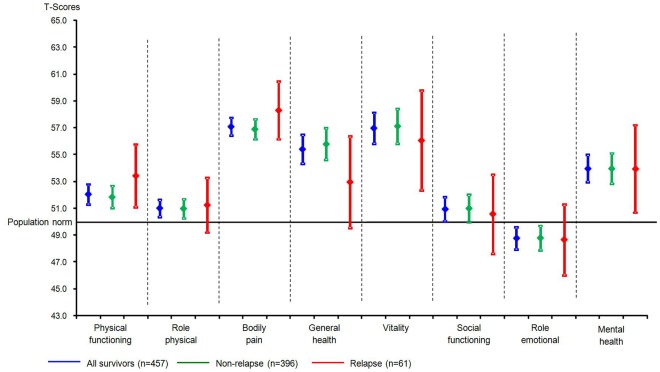
Short Form-36 scales, in all survivors and by relapse status, adjusted results. Full model, adjusted for gender, current age, time since diagnosis, having a partner, education, chemo−/radiotherapy, bone marrow transplantation, duration of therapy, and self-reported late effects; German population norm used. SF-36: Short Form-36.

In multivariable analysis, the difference in general health between survivors with and without relapse remained statistically significant in baseline, social and therapy models (51.1 vs. 55.9, p=0.003; 51.2 vs. 55.8, p=0.004; 56.0 vs. 50.8, p=0.008) (**Table S2**). However, when we adjusted for self-reported late effects, the difference in SF-36 scales disappeared (“general health” in late effects model: 53.9 vs. 55.4, p=0.359; “general health” in full model: 53.0 vs. 55.8, p=0.135; **Figure 2**).

We looked at the distribution of the answers aggregated into “general health” in order to better understand the difference. Fewer survivors of relapsed ALL chose the statements, “I am as healthy as anybody I know,” and, “My health is excellent,” than survivors of non-relapsed ALL (38% vs. 58%, p=0.006; and 39% vs. 55%, p=0.004 respectively) (**Table 3**).

**Table 3:** Distribution of the answers to items of the general health scale, overall and by relapse status.

		All (n=457)		Relapse (n=61)			Non-relapse (n=396)			p
		N	%	n	%	95CI	n	%	95CI	
**I seem to get sick a little easier than other people**	definitively true	23	5.1	4	6.7	0.2 – 13.2	19	4.8	2.7 – 7.0	0.051
	mostly true	47	10.4	7	11.7	3.3 – 20.0	40	10.2	7.2 – 13.2	
	don’t know	49	10.8	10	16.7	7.0 – 26.4	39	9.9	7.0 – 12.9	
	mostly false	99	21.9	15	25.0	13.7 – 36.3	84	21.4	17.3 – 25.4	
	definitively false	235	51.9	24	40.0	27.2 – 52.8	211	53.7	48.7 – 58.6	
**I am as healthy as anybody I know**	definitively true	249	55.1	22	36.7	24.1 – 49.2	227	57.9	53.0 – 62.8	0.006
	mostly true	119	26.3	23	38.3	25.7 – 51.0	96	24.5	20.2 – 28.8	
	don’t know	34	7.5	7	11.7	3.3 – 20.0	27	6.9	4.4 – 9.4	
	mostly false	34	7.5	7	11.7	3.3 – 20.0	27	6.9	4.4 – 9.4	
	definitively false	16	3.5	1	1.7	−1.7 – 5.0	15	3.8	1.9 – 5.7	
**I expect my health to get worse**	definitively true	3	0.7	1	1.7	−1.7 – 5.1	2	0.5	−0.2 – 1.2	0.045
	mostly true	16	3.6	4	6.8	0.2 – 13.4	12	3.1	1.4 – 4.8	
	don’t know	63	14.0	12	20.3	9.8 – 30.9	51	13.1	9.7 – 16.4	
	mostly false	65	14.5	8	13.6	4.6 – 22.6	57	14.6	11.1 – 18.1	
	definitively false	302	67.3	34	57.6	44.6 – 70.6	268	68.7	64.1 – 73.3	
**My health is excellent**	definitively true	239	53.0	23	39.0	26.2 – 51.8	216	55.1	50.2 – 60.0	0.004
	mostly true	151	33.5	20	33.9	21.5 – 46.3	131	33.4	28.7 – 38.1	
	don’t know	26	5.8	7	11.9	3.4 – 20.4	19	4.8	2.7 – 7.0	
	mostly false	21	4.7	7	11.9	3.4 – 20.4	14	3.6	1.7 – 5.4	
	definitively false	14	3.1	2	3.4	−1.4 – 8.1	12	3.1	1.3 – 4.8	

Abbreviation: 95CI, 95% confidence interval.

### Sensitivity Analysis

Repeated analyses with French and Italian norm population HRQOL produced similar results, both in univariable and in multivariable regressions (**Tables S3 and S4**).

## Discussion

We found that ALL survivors, on average, reported HRQOL similar to the norm population, even after a relapse. Survivors of relapsed ALL perceived their general health to be lower than did non-relapsed ALL survivors. This difference became insignificant when we adjusted for late effects, indicating that late effects are a major underlying reason for the lower HRQOL in relapsed survivors.

### Strengths and Limitations of the Present Study

A major strength of our study is the focus on relapse in ALL survivors. Most previous studies mixed all relapses across diagnostic groups. This was a problem because relapse is defined differently and leads to different treatment modifications. Large sample size, high response rate and the population-based nature of our study make it stronger than most other studies that investigated the role of relapse on HRQOL in childhood cancer survivors (**Table 4**).

**Table 4:** Comparison of papers on the association of childhood cancer relapse with HRQOL.

	Cohort	Sample Size	Type of Cancer	Measurement Tool	Multivariable result	Independent variables in multivariable regressions
Present paper	Population basedSwiss ChildhoodCancer SurvivorStudy	396 N-R, 61 R	ALL	SF-36	Survivors of relapsed andnon-relapsed ALL had similarHRQOL, except in generalhealth. In regression analysis,this difference wasexplained by late effects.	Gender, age, time since diagnosis, having a partner, education, chemo−/radiotherapy, bone marrow transplantation, duration of therapy, self- reported late effects and relapse
Stam and colleagues (2006) [14]	Attendants of Dutch long-term follow-up clinic	310 N-R, 43 R	mixed	SF-36	Relapse did not contributeto HRQOL.	Gender, age, diagnosis, treatment, age at first diagnosis, duration of treatment, and “relapse or second malignancy”
Zebrack and Chesler (2002) [15]	Former patients of a “mid-western children’s hospital”	160 N-R, 15 R	mixed	Quality ofLife-CancerSurvivors	Relapse did not contributeto HRQOL.	Gender, age, parent income, living arrangement, diagnosis, medical condition, age at diagnosis, after-effects reported and relapse
Maunsell andcolleagues (2006) [16]	Canadian Childhood Cancer Surveillance and ControlProgram	1178 N-R, 156 R	mixed	SF-36, self-esteem and optimismscales, satisfactionwith life scale	More than one treatmentseries (as a proxy for relapse)was independently associatedwith poorer HRQOL inthe physical dimensions.	“CNS or bone cancer”, two organs with dysfunction, “all three treatment modalities” (surgery, chemo-, and radiotherapy), cranial radiation, more than one treatment series
Harila and colleagues (2010) [17]	Hospital-based studyin Oulu/Finland	63 N-R, 11 R	ALL	SF-36	−	−

Abbreviations: N-R, Survivors of non-relapsed childhood cancer; R, Survivors of relapsed childhood cancer; ALL, acute lymphoblastic leukemia; SF-36, Short Form-36; HRQOL, health-related quality of life; CNS, central nervous system.

Our study also has limitations. Due to limited statistical power we could not look at different subgroups of relapses (late or early, isolated bone marrow or extramedullary relapses, combined relapses), nor could we distinguish between different subgroups of late effects. We did not consider the exact drugs and cumulative doses used in treatment, nor the sites of radiation. Finally, no SF-36 norm data is available for Switzerland.

### Comparison with Other Studies

Previous studies comparing HRQOL of ALL survivors with population norms found similar results. Zeltzer and colleagues (2009) compared ALL survivors with US norms and siblings [22]. Survivors had lower SF-36 means for physical function, role physical, role emotional, and vitality than the norm population, but they were still well within one standard deviation of the norm. In a study by Reulen and colleagues (2007) used the SF-36 physical and mental summary measures to compare 2558 leukemia survivors with British norms and found no difference [20]. Neither was a difference found between ALL survivors and their control relatives when Short Form-12 (SF-12) was used [21].

In Zebrack and Chesler’s study (2002), relapse did not contribute to HRQOL, even when late effects were adjusted for [15]. The latter supports our findings, but a Canadian study found that more than one treatment series (as a proxy for relapse) was independently associated with poorer quality of life in the physical dimensions, even when major late effects were adjusted for [16]. In contrast, a Finnish study found higher scores for vitality and mental health in relapsed ALL survivors compared to healthy controls [17]. However, the analysis was not adjusted.

### Possible Explanations and Implications

Several earlier studies attempted to explain the rather surprising result that childhood cancer survivors often report similar or better HRQOL than general populations. Survivors’ subjective perception of HRQOL may be affected by a desire to be “as normal as possible,” causing a response shift (a change in the meaning of one’s self-evaluation of quality of life) [36]. Caught in the “paradox of satisfaction [38],” childhood cancer survivors also tend to deny difficulties on QOL measures [37] and to report high QOL even under difficult living conditions. But surviving childhood cancer may also result in post-traumatic growth or thriving [39], [40], suggesting that survivors may indeed experience high QOL despite problems.

Relapsed and non-relapsed ALL survivors reported on similar HRQOL, except on one SF-36 scale. In relapsed patients, general health was significantly poorer, very likely as a consequence of late effects associated with relapse. As it was described in earlier studies, those late effects can become severe; therefore, our results have implications for follow-up care: We encourage specialists who conduct follow-up to screen for poor general health in survivors after a relapse and, when appropriate, specifically seek and treat underlying late effects.

### Open Questions and Future Research

The small effect we found in the present study requires in-depth investigation in larger groups of patients. Treatment intensity, adjuvant radiotherapy and stem cell transplantation with myeloablative regimens may induce a series of somatic and mental late chronic conditions after a relapse almost unknown after first-line treatments for pediatric ALL. We should attempt to better describe and quantify late effects by promoting regular long-term follow-up visits, where survivors can be examined and interviewed. Further preventing and minimizing late effects will help to improve quality of life in survivors of ALL, particularly those who had experienced a relapse.

## Supporting Information

Table S1 **SF-36 scales explained.**
(DOCX)

Table S2 **Effect of relapse on SF-36 scales: Additional adjusted results (social, therapy, and late effects model).**
(DOCX)

Table S3 **Sensitivity analyses - Effect of relapse on SF-36 scales (French norm, uni- and multivariable).**
(DOCX)

Table S4 **Sensitivity analyses - Effect of relapse on SF-36 scales (Italian norm, uni- and multivariable).**
(DOCX)
