# Supplementary material for: Health-Related Quality of Life in Long-Term Survivors of Relapsed Childhood Acute Lymphoblastic Leukemia
Source: PLoS One. 2012 May 25;7(5):e38015. doi: 10.1371/journal.pone.0038015 (PMC3360640; doi:10.1371/journal.pone.0038015)
Supplement: Table S1 — SF-36 scales explained. (DOCX) [file pone.0038015.s001.docx]

**Table S1. SF-36 scales explained**

| **SF-36 Scale** | **Definition of lowest possible score** | **Definition of highest possible score** |
| --- | --- | --- |
| Physical Functioning | Very limited in performing all physical activities, including bathing or dressing | Performs all types of physical activities including the most vigorous without limitations due to health |
| Role Physical | Problems with work or other daily activities as a result of physical health | No problems with work or other daily activities |
| Bodily Pain | Very severe and extremely limiting pain | No pain or limitations due to pain |
| General Health | Evaluates personal health as poor and believes it is likely to get worse | Evaluates personal health as excellent |
| Vitality | Feels tired and worn out all of the time | Feels full of pep and energy all of the time |
| Social Functioning | Extreme and frequent interference with normal social activities due to physical and emotional problems | Performs normal social activities without interference due to physical or emotional problems |
| Role Emotional | Problems with work or other daily activities as a result of emotional problems | No problems with work or other daily activities |
| Mental Health | Feelings of nervousness and depression all of the time | Feels peaceful, happy, and calm all of the time |

Abbreviation: SF-36, Short Form-36. Source: http://www.sf-36.org/tools/sf36.shtml#VERS2 (website visited: 11 Oct 2011; used with kind permission of John E. Ware, Jr.)
