# Supplementary material for: Health-Related Quality of Life in Long-Term Survivors of Relapsed Childhood Acute Lymphoblastic Leukemia
Source: PLoS One. 2012 May 25;7(5):e38015. doi: 10.1371/journal.pone.0038015 (PMC3360640; doi:10.1371/journal.pone.0038015)
Supplement: Table S2 — Effect of relapse on SF-36 scales: Additional adjusted results (social, therapy, and late effects model). (DOCX) [file pone.0038015.s002.docx]

**Table S2. Effect of relapse on SF-36 scales: Additional adjusted results (social, therapy, and late effects model)**

|  |  | **Adjusted, social model^a^** | | | |  | **Adjusted, therapy model^b^** | | | |  | **Adjusted, late effects model^c^** | | | |  |
| --- | --- | --- | --- | --- | --- | --- | --- | --- | --- | --- | --- | --- | --- | --- | --- | --- |
|  |  | **All (n=457)** | **Non-relapse (n=396)** | **Relapse (n=61)** | **p^d^** |  | **All (n=457)** | **Non-relapse (n=396)** | **Relapse (n=61)** | **p^d^** |  | **All (n=457)** | **Non-relapse (n=396)** | **Relapse (n=61)** | **p^d^** |  |
| **PF** |  |  |  |  |  |  |  |  |  |  |  |  |  |  |  |  |
| Mean |  | 51.9 | 52.1 | 50.7 | 0.214 |  | 52.0 | 51.9 | 52.2 | 0.841 |  | 52.0 | 51.9 | 52.3 | 0.690 |  |
| 95CI |  | [51.2-52.7] | [51.3-52.9] | [48.7-52.8] |  |  | [51.2-52.7] | [51.1-52.7] | [49.8-54.6] |  |  | [51.3-52.7] | [51.1-52.7] | [50.3-54.4] |  |  |
| **RP** |  |  |  |  |  |  |  |  |  |  |  |  |  |  |  |  |
| Mean |  | 51.0 | 51.2 | 49.8 | 0.141 |  | 50.9 | 51.1 | 49.9 | 0.310 |  | 50.9 | 50.9 | 51.3 | 0.662 |  |
| 95CI |  | [50.3-51.6] | [50.5-51.9] | [48.0-51.5] |  |  | [50.3-51.6] | [50.4-51.8] | [47.8-52.0] |  |  | [50.3-51.5] | [50.2-51.5] | [49.5-53.1] |  |  |
| **BP** |  |  |  |  |  |  |  |  |  |  |  |  |  |  |  |  |
| Mean |  | 57.2 | 57.2 | 57.1 | 0.941 |  | 57.1 | 57.2 | 56.9 | 0.793 |  | 57.1 | 56.9 | 58.4 | 0.137 |  |
| 95CI |  | [56.5-57.9] | [56.5-57.9] | [55.3-59.0] |  |  | [56.5-57.8] | [56.4-57.9] | [54.6-59.1] |  |  | [56.5-57.8] | [56.2-57.6] | [56.6-60.3] |  |  |
| **GH** |  |  |  |  |  |  |  |  |  |  |  |  |  |  |  |  |
| Mean |  | 55.2 | 55.8 | 51.2 | 0.004 |  | 55.3 | 56.0 | 50.8 | 0.008 |  | 55.2 | 55.4 | 53.9 | 0.359 |  |
| 95CI |  | [54.1-56.3] | [54.6-56.9] | [48.3-54.1] |  |  | [54.2-56.3] | [54.8-57.1] | [47.3-54.3] |  |  | [54.2-56.2] | [54.3-56.5] | [51.0-56.9] |  |  |
| **VT** |  |  |  |  |  |  |  |  |  |  |  |  |  |  |  |  |
| Mean |  | 56.8 | 57.1 | 54.7 | 0.170 |  | 57.0 | 57.5 | 53.4 | 0.053 |  | 57.0 | 56.8 | 58.2 | 0.408 |  |
| 95CI |  | [55.6-58.0] | [55.9-58.4] | [51.5-58.0] |  |  | [55.8-58.1] | [56.2-58.8] | [49.6-57.2] |  |  | [55.9-58.1] | [55.6-58.0] | [55.0-61.4] |  |  |
| **SF** |  |  |  |  |  |  |  |  |  |  |  |  |  |  |  |  |
| Mean |  | 50.9 | 51.1 | 49.6 | 0.272 |  | 50.9 | 51.3 | 48.3 | 0.081 |  | 50.9 | 50.7 | 52.0 | 0.354 |  |
| 95CI |  | [50.0-51.8] | [50.1-52.1] | [47.0-52.1] |  |  | [50.0-51.8] | [50.3-52.3] | [45.3-51.4] |  |  | [50.0-51.7] | [49.7-51.6] | [49.4-54.5] |  |  |
| **RE** |  |  |  |  |  |  |  |  |  |  |  |  |  |  |  |  |
| Mean |  | 48.8 | 49.0 | 47.4 | 0.185 |  | 48.8 | 49.1 | 47.2 | 0.187 |  | 48.9 | 48.8 | 49.2 | 0.737 |  |
| 95CI |  | [48.0-49.6] | [48.1-49.9] | [45.1-49.7] |  |  | [48.0-49.6] | [48.2-50.0] | [44.5-49.8] |  |  | [48.1-49.6] | [48.0-49.6] | [46.9-51.5] |  |  |
| **MH** |  |  |  |  |  |  |  |  |  |  |  |  |  |  |  |  |
| Mean |  | 53.9 | 54.1 | 53.2 | 0.560 |  | 54.0 | 54.3 | 51.9 | 0.196 |  | 54.0 | 53.7 | 55.5 | 0.244 |  |
| 95CI |  | [52.9-55.0] | [53.0-55.1] | [50.4-56.0] |  |  | [53.0-55.0] | [53.2-55.4] | [48.7-55.2] |  |  | [53.0-54.9] | [52.7-54.8] | [52.7-58.3] |  |  |

Abbreviations: PF, Physical functioning; RF, Role physical; BP, Bodily pain; GH, General health; VT, Vitality; SF, Social functioning; RE, Role emotional; MH, Mental health; SF-36, Short Form-36; 95CI, 95% confidence interval.

^a^Social model: adjusted for gender, current age, time since diagnosis, having a partner, education.
^b^Therapy model: adjusted for gender, current age, time since diagnosis, chemo-/radiotherapy, bone marrow transplantation, duration of therapy.
^c^Late effects model: adjusted for gender, current age, time since diagnosis, self-reported late effects.
^d^p-values calculated from likelihood-ratio tests.
