# Supplementary material for: Health-Related Quality of Life in Long-Term Survivors of Relapsed Childhood Acute Lymphoblastic Leukemia
Source: PLoS One. 2012 May 25;7(5):e38015. doi: 10.1371/journal.pone.0038015 (PMC3360640; doi:10.1371/journal.pone.0038015)
Supplement: Table S3 — Sensitivity analyses - Effect of relapse on SF-36 scales (French norm, uni- and multivariable). (DOCX) [file pone.0038015.s003.docx]

**Table S3. Sensitivity analyses - Effect of relapse on SF-36 scales (French norm, uni- and multivariable)**

|  |  |  | **Unadjusted** | | |  | **Adjusted, full model^a^** | | | |
| --- | --- | --- | --- | --- | --- | --- | --- | --- | --- | --- |
|  | **All  (n=457)** |  | **Non-relapse  (n=396)** | **Relapse  (n=61)** | **p** |  | **All  (n=457)** | **Non-relapse  (n=396)** | **Relapse  (n=61)** | **p^b^** |
| **Physical functioning** |  |  |  |  |  |  |  |  |  |  |
| Mean | 55.1 |  | 55.3 | 54.3 | 0.037 |  | 55.2 | 55.1 | 55.8 | 0.336 |
| 95CI | 54.8-55.5 |  | 54.9-55.7 | 53.1-55.5 |  |  | 54.8-55.6 | 54.7-55.5 | 54.5-57.0 |  |
| **Role physical** |  |  |  |  |  |  |  |  |  |  |
| Mean | 53.9 |  | 54.0 | 53.0 | 0.113 |  | 54.0 | 53.9 | 54.0 | 0.927 |
| 95CI | 53.5-54.3 |  | 53.6-54.4 | 51.8-54.2 |  |  | 53.6-54.3 | 53.5-54.4 | 52.8-55.2 |  |
| **Bodily pain** |  |  |  |  |  |  |  |  |  |  |
| Mean | 56.4 |  | 56.5 | 56.4 | 0.921 |  | 56.4 | 56.2 | 57.5 | 0.271 |
| 95CI | 55.8-57.1 |  | 55.8-57.1 | 54.6-58.2 |  |  | 55.8-57.0 | 55.5-56.9 | 55.4-59.5 |  |
| **General health** |  |  |  |  |  |  |  |  |  |  |
| Mean | 56.0 |  | 56.5 | 53.0 | 0.002 |  | 56.1 | 56.4 | 54.1 | 0.110 |
| 95CI | 55.2-56.8 |  | 55.6-57.3 | 50.6-55.3 |  |  | 55.3-56.9 | 55.5-57.3 | 51.5-56.7 |  |
| **Vitality** |  |  |  |  |  |  |  |  |  |  |
| Mean | 52.8 |  | 53.0 | 51.3 | 0.182 |  | 52.8 | 52.9 | 52.2 | 0.617 |
| 95CI | 52.0-53.6 |  | 52.2-53.8 | 48.9-53.7 |  |  | 52.1-53.6 | 52.1-53.8 | 49.8-54.7 |  |
| **Social functioning** |  |  |  |  |  |  |  |  |  |  |
| Mean | 54.0 |  | 54.1 | 53.0 | 0.494 |  | 54.0 | 54.1 | 53.8 | 0.836 |
| 95CI | 53.2-54.7 |  | 53.4-54.9 | 50.6-55.3 |  |  | 53.3-54.7 | 53.3-54.9 | 51.5-56.1 |  |
| **Role emotional** |  |  |  |  |  |  |  |  |  |  |
| Mean | 52.5 |  | 52.6 | 51.9 | 0.499 |  | 52.5 | 52.5 | 52.5 | 0.949 |
| 95CI | 52.1-53.0 |  | 52.1-53.1 | 50.4-53.4 |  |  | 52.0-53.0 | 52.0-53.1 | 50.9-54.0 |  |
| **Mental health** |  |  |  |  |  |  |  |  |  |  |
| Mean | 54.2 |  | 54.3 | 53.6 | 0.638 |  | 54.2 | 54.2 | 54.2 | 0.991 |
| 95CI | 53.5-54.9 |  | 53.5-55.1 | 51.6-55.7 |  |  | 53.5-55.0 | 53.4-55.0 | 51.9-56.6 |  |

Abbreviations: SF-36, Short Form-36; 95CI, 95% confidence interval.

^a^Full model: adjusted for gender, current age, time since diagnosis, having a partner, education, chemo- /radiotherapy, bone marrow transplantation, duration of therapy, and self-reported late effects.

^b^p-values calculated from likelihood-ratio tests.
