# Supplementary material for: Health-Related Quality of Life in Long-Term Survivors of Relapsed Childhood Acute Lymphoblastic Leukemia
Source: PLoS One. 2012 May 25;7(5):e38015. doi: 10.1371/journal.pone.0038015 (PMC3360640; doi:10.1371/journal.pone.0038015)
Supplement: Table S4 — Sensitivity analyses - Effect of relapse on SF-36 scales (Italian norm, uni- and multivariable). (DOCX) [file pone.0038015.s004.docx]

**Table S4. Sensitivity analyses - Effect of relapse on SF-36 scales (Italian norm, uni- and multivariable)**

|  |  |  | **Unadjusted** | | | **Adjusted, full model^a^** | | |  |
| --- | --- | --- | --- | --- | --- | --- | --- | --- | --- |
|  | **All  (n=457)** |  | **Non-relapse  (n=396)** | **Relapse  (n=61)** | **p** | **All  (n=457)** | **Non-relapse  (n=396)** | **Relapse  (n=61)** | **p^b^** |
| **Physical functioning** |  |  |  |  |  |  |  |  |  |
| Mean | 55.3 |  | 55.4 | 54.5 | 0.037 | 55.3 | 55.2 | 55.8 | 0.336 |
| 95CI | 54.9-55.6 |  | 55.0-55.7 | 53.5-55.6 |  | 55.0-55.6 | 54.8-55.6 | 54.7-56.8 |  |
| **Role physical** |  |  |  |  |  |  |  |  |  |
| Mean | 54.3 |  | 54.4 | 53.5 | 0.113 | 54.4 | 54.4 | 54.4 | 0.927 |
| 95CI | 54.0-54.7 |  | 54.1-54.8 | 52.4-54.6 |  | 54.0-54.7 | 53.9-54.8 | 53.3-55.6 |  |
| **Bodily pain** |  |  |  |  |  |  |  |  |  |
| Mean | 56.1 |  | 56.1 | 56.0 | 0.921 | 56.1 | 55.9 | 57.0 | 0.271 |
| 95CI | 55.5-56.7 |  | 55.5-56.7 | 54.4-57.7 |  | 55.5-56.6 | 55.3-56.6 | 55.2-58.9 |  |
| **General health** |  |  |  |  |  |  |  |  |  |
| Mean | 57.4 |  | 57.8 | 54.5 | 0.002 | 57.4 | 57.7 | 55.6 | 0.110 |
| 95CI | 56.6-58.1 |  | 57.0-58.6 | 52.3-56.7 |  | 56.7-58.2 | 56.9-58.6 | 53.1-58.0 |  |
| **Vitality** |  |  |  |  |  |  |  |  |  |
| Mean | 52.5 |  | 52.7 | 51.1 | 0.182 | 52.5 | 52.6 | 52.0 | 0.617 |
| 95CI | 51.8-53.3 |  | 51.9-53.5 | 48.7-53.4 |  | 51.8-53.3 | 51.8-53.5 | 49.6-54.4 |  |
| **Social functioning** |  |  |  |  |  |  |  |  |  |
| Mean | 55.3 |  | 55.4 | 54.3 | 0.494 | 55.3 | 55.4 | 55.1 | 0.836 |
| 95CI | 54.6-56.0 |  | 54.7-56.2 | 52.0-56.6 |  | 54.6-56.1 | 54.6-56.2 | 52.9-57.4 |  |
| **Role emotional** |  |  |  |  |  |  |  |  |  |
| Mean | 53.6 |  | 53.7 | 53.0 | 0.499 | 53.6 | 53.6 | 53.5 | 0.949 |
| 95CI | 53.2-54.0 |  | 53.2-54.2 | 51.6-54.4 |  | 53.1-54.0 | 53.1-54.1 | 52.1-55.0 |  |
| **Mental health** |  |  |  |  |  |  |  |  |  |
| Mean | 54.6 |  | 54.6 | 54.0 | 0.638 | 54.6 | 54.6 | 54.6 | 0.991 |
| 95CI | 53.9-55.2 |  | 53.9-55.4 | 52.1-55.9 |  | 53.9-55.2 | 53.8-55.3 | 52.4-56.8 |  |

Abbreviations: SF-36, Short Form-36; 95CI, 95% confidence interval.

^a^Full model: adjusted for gender, current age, time since diagnosis, having a partner, education, chemo- /radiotherapy, bone marrow transplantation, duration of therapy, and self-reported late effects.

^b^p-values calculated from likelihood-ratio tests.
